# Cases of Extra-Uterine Pregnancy and Pyosalpinx Reported to Atlanta Society of Medicine

**Published:** 1893-05

**Authors:** R. R. Kime

**Affiliations:** Professor Gynecology Atlanta Polyclinic, Lecturer on Gynecology Southern Medical College, Atlanta, Ga.


### CASES OF EXTRA-UTERINE PREGNANCY AND PYOSALPINX REPORTED TO ATLANTA SOCIETY OF MEDICINE.

BY R. R. KIME, M. D.,
Professor Gynecology Atlanta Polyclinic, Lecturer on Gynecology Southern Medical College, Atlanta, Ga.

CASE 1.—S., colored, age twenty-six, married ten years, first flow at thirteen years, always regular as to time, but suffered severe pain during flow. No children, no miscarriages, general health good; had been treated for uterine trouble, cervix dilated several years previously.

Last flow second week in October, 1892. November 22d Dr. J. H. Green, of Decatur, was called, found patient with usual symptoms of pregnancy, some pain and tenderness in pelvic region, with enlargement in right broad ligament close to uterus. Last week in December Dr. Mason was called in absence of Dr. Green. Patient had been suddenly seized with pain in bowels, sick stomach, collapse, hemorrhage, the clots of which soaked in water left behind a membrane only. The doctor diagnosed miscarriage and prescribed opiates, rest, etc. After this date symptoms of pregnancy suddenly disappeared except enlargement in pelvis which continued to increase in size to date of operation accompanied with pain, tenderness, occasional hemorrhages and difficulty in urinating, all of which were present until operated upon.

Saw her first February 14, 1893. One week previous she had a chill. After chill had elevation of temperature reaching as high as $102\frac{1}{5}°$. Pulse ranging from 90 to 120. Examination revealed tumor in pelvis and lower abdomen size of four or five months pregnancy. From clinical history and physical signs diagnosed extra-uterine pregnancy, primarily in right tube, with rupture extra peritoneally into broad ligament, uterus being crowded upwards anteriorly to left of median line. Advised counsel and operation. Drs. W. A. Crow and C. S. Webb being called next day concurred in diagnosis. Sound introduced into uterus measuring two and a half inches.

The patient was removed with care to Atlanta Polyclinic, where she was operated upon February 20th, with the able assistance of Drs. Crow, J. C. Avary, Joe H. Green and others of the Polyclinic staff, ether being used.

Median incision enlarged from pubes to umbilicus. Parietal peritoneum thickened at points adherent. First cyst opened contained about one ounce pus, the second two or three ounces thin fluid resembling that of an ovarian cystoma; cutting on through a layer of vascular tissue a third cyst was opened containing a pint of dark bloody fluid, after which the fourth cyst was ruptured in which fœtus was found partially decomposed, measuring three and a half inches in length. The cyst and abdomen were thoroughly irrigated with Thiersch's solution and sterilized water, glass drainage tubes inserted to bottom of cyst after cyst wall was sutured to parietal peritoneum, peritoneal cavity closed and wound dressed, leaving three artery clamps attached to cyst wall near placenta to check bleeding, as all efforts to apply ligatures increased the hemorrhage by cutting through the vascular tissue. Forceps removed forty-eight hours after operation, no hemorrhage. Not any suppuration for seven days after operation; on the seventh day removed glass tube, and put in two rubber tubes. The temperature that day reached $103\frac{3}{5}$°, the highest temperature previously being $101\frac{1}{5}$° Fah., the day previous 101°. Replaced glass tube, irrigated cyst, after which temperature subsided and has ranged from 99° to $100\frac{2}{5}$° since. Patient well nourished, secretions good, with discharge from cyst to present date. It is difficult to keep up good drainage on account of a tendency of canal to close where band of tissue had to be separated to enter third cyst; using rubber drainage tubes for last two weeks.

*Remarks.*—This case could not have been relieved by elytrotomy successfully, for two cysts, one containing pus, could not have been drained; for same reason nature could not have gotten rid of the trouble, besides the septic condition present demanded operative interference.

It serves to illustrate the necessity for operative measures by abdominal incision and not trust to the ineffectual efforts of nature or less satisfactory methods of electricity, vaginal incision, etc.

CASE 2.—Referred by Dr. Elkin; colored, age thirty-four years, married eight years, no children, no miscarriages, first flow at fourteen years, regular since except at times, lasting two weeks or more with menorrhagia, with but little pain except at times of hemorrhage or during attacks of pelvic peritonitis, having had four or five attacks.

Saw her first January, 1892; found retroversion of uterus fixed by adhesion, pyosalpinx. So diagnosed, the left tube not so much enlarged as right. Refused operation but improved on tonics, iron and mercuric chloride internally, with local applications, iodine, glycerine tampons and hot douches. Passed from under my care until December 19th, 1892; called by request of specialist under whose care she had been for some five or six days, and by request of physician and patient took charge of case.

She then had a temperature of $105\frac{2}{5}°$; pulse 130; bowels tympanitic, tender; two chills the night previous; local examination revealed inflammatory deposits in both. Broad ligaments, tubes enlarged with retroversion and fixation of uterus, with evidences of absorption of purulent material. Gave

Quinine, 5 gr.

Calomel, $\frac{1}{4}$ gr.

Phenacetine, 1 gr.

Every two hours. Salines.

Fluid extract nux vomica, liquor sedans, tr. cinchona comp., liquid pepsin, good nourishment, with hot vaginal douches; temperature subsided, chills checked, patient improved.

The temperature ranged from 99° to $105\frac{2}{5}°$ until tubes were removed March 11, 1893, a period of about three months, although the pelvic condition improved materially by use of iodine applications, boroglyceride tampons and hot vaginal douches; with the internal use of iron, mercuric chloride and hydrochloric acid internally.

Patient etherized; operated March 11, 1893, assisted by Drs. Crow, Avary, Hurt, Elkin and others.

Both tubes and uterus were adherent to other tissues and broad ligaments congenitally short, rendering it impossible to bring them to abdominal incision for ligation; had to place patient in Trendelenburg posture and tie them off down in pelvis. On account of the length of time consumed in separating and ligating, with condition of patient, the left ovary was left, being adherent, lower in broad ligament, very difficult to reach. A glass drainage tube was used; wound closed in the usual manner.

Everything progressed nicely for thirty-six hours, when a hemorrhage occurred, patient became almost pulseless, ranging at about 140 to 160; sick at stomach, fainty, anxious countenance, by posture injecting hot water into tube, checked it.

Drainage tube kept in four days, then removed. Patient's temperature had gradually subsided and is normal last two days, the first time so long in three or four months.

